# Comparability of off the shelf foot orthoses in the redistribution of forces in midfoot osteoarthritis patients

**DOI:** 10.1016/j.gaitpost.2016.07.012

**Published:** 2016-09

**Authors:** Graham J. Chapman, Jill Halstead, Anthony C. Redmond

**Affiliations:** a Institute of Rheumatic and Musculoskeletal Disease, University of Leeds, Leeds, UK; b NIHR Leeds Musculoskeletal Biomedical Research Unit, Leeds, UK; c Arthritis Research UK, Experimental Osteoarthritis Treatment Centre, UK; d Arthritis Research UK, Sports, Exercise and Osteoarthritis Centre, UK

**Keywords:** Foot, Osteoarthritis, Orthoses, Midfoot, Plantar pressure, Sham device

## Abstract

**Background:**

Midfoot osteoarthritis (OA) is more prevalent and strongly associated with pain than previously thought. Excessive mechanical loading of the midfoot structures may contribute to midfoot OA and studies suggest that functional foot orthoses (FFO) may relieve pain through improving function. This exploratory study aimed to evaluate the mechanical effect of two off-the-shelf FFOs, compared to a sham orthosis in people with midfoot OA.

**Methods:**

Thirty-three participants with radiographically confirmed symptomatic midfoot OA were randomly assigned to wear either a commercially available FFO or a sham orthosis. After wearing their assigned orthoses for 12 weeks, plantar pressure measurements were obtained under shoe-only and assigned orthoses conditions. Participants assigned to the sham, were additionally tested wearing a second type of FFO at the end of trial. Descriptive mean change (±95% confidence intervals) in plantar pressure for each orthoses condition, versus a shoe only baseline condition are presented.

**Findings:**

Compared to the shoe only conditions, both FFOs decreased hindfoot and forefoot maximum force and peak pressure, whilst increasing maximum force and contact area under the midfoot. The sham orthosis yielded plantar pressures similar to the shoe-only condition.

**Interpretation:**

Findings suggest that both types of off-the-shelf FFO may provide mechanical benefit, whilst the sham orthoses produced similar findings to the shoe only condition, indicating appropriate sham properties. This paper provides insight into the mechanisms of action underpinning the use of FFOs and sham orthoses, which can inform future definitive RCTs examining the effect of orthoses on midfoot OA.

## Highlights

• Both types of functional foot orthoses produce similar mechanical effects compared to the sham orthosis.
• The sham orthosis was comparable to the shoe only condition.
• Both functional foot orthoses and the sham orthoses are appropriate for use in future RCTs.

## 1 Introduction

Foot pain is a common problem, affecting between 20%–42% of adults aged 45 years and older [1], [2], [3], and limits activities of daily living [1], [2], [4], [5], [6], [7], [8]. Recent studies using a radiographic foot atlas [9], demonstrated midfoot OA is more prevalent and more strongly associated with pain than thought previously [10], [11], [12], [13]. In the UK, 16% of people over 50 years old suffer from painful radiographic foot OA, which commonly affects midfoot joints [14]. Midfoot OA has been shown to alter foot posture causing significantly higher forces and plantar pressures acting on the midfoot than people without midfoot OA [15], [16]. These plantar pressure differences also correlate moderately with pain [16], suggesting anatomical and/or biomechanical factors may contribute to the development of midfoot OA.

Foot orthoses are a common conservative treatment for many musculoskeletal problems [17], [18], [19], intended to alleviate pain and improve function. In people with midfoot OA, short-term non-randomised studies demonstrated functional foot orthoses (FFO) improve pain and function [20], [21]. Similarly, a recent feasibility study demonstrated that midfoot OA participants randomly assigned to the FFO group reported significantly greater improvements in clinical and functional outcomes compared to a sham intervention group [22]. Taken together, these findings suggest increased forces and/or pressures acting on the midfoot may contribute to increased mechanical loading on joints, and FFOs may support these structures thereby reducing pain and improving function. In accordance with recently published recommendations on conducting trials examining treatment devices for OA [23], the aim of this exploratory study was to investigate the mechanism of action of two different off-the-shelf FFOs with differing properties; a firmer (shore 50) more controlling device (FFO A) and a softer (shore 35) more cushioning device (FFO B) compared to a sham device. Evaluating differences in plantar pressures when wearing either FFO or sham orthoses, in people with radiographically confirmed midfoot OA will provide objective information for designing/choosing an appropriate orthosis/sham for future RCTs.

## 2 Methods

Participants with foot pain were recruited from community musculoskeletal and podiatry services to participate in a feasibility trial testing foot orthoses as a treatment for midfoot OA. Participants were included if they were aged 18 years and older; reported localised midfoot pain for over three months using a standardised foot pain map [4]; reported midfoot pain when weight-bearing; and had evidence of radiographic OA in at least one of the following: talo-navicular joint, naviculo-medial cuneiform joint, cuneiform-first metatarsal joint, cuneiform-second metatarsal joint. A musculoskeletal radiologist verified all radiographs, defining the presence of OA in the relevant joints by a score of two or higher for either osteophytes or joint space narrowing, from either the dorso-plantar or lateral views according to a previously developed foot atlas [9]. Exclusion criteria included any lower limb orthopaedic surgery within the past 12 months, inflammatory joint disease, sensory neuropathy of the feet (insensate to 10 g monofilament at any of the 10 sites on the foot), radiographically evident stress fractures or a history of any clinically significant disease or major disorder that would not be conducive to study participation. Other exclusion criteria were the inability to undergo x-ray examination for medical reasons, inability to complete the gait analysis or current wearing of prescribed or off-the-shelf contoured or cast orthoses. Only the symptomatic foot was tested in this study. For bilateral OA participants, the more painful foot was defined as the study foot. If foot pain was equal in both feet, the study foot was defined by the participant’s dominant foot as determined by the first step technique. Research Ethics approval was obtained for the study and all participants provided written informed consent prior to commencing the study.

### 2.1 Interventions

The present mechanism of action sub-study was nested within a 12 week, double-blind, two-arm parallel group randomised controlled feasibility study reported elsewhere [22], examining the effects of FFOs on symptomatic midfoot OA. Participants were randomly assigned to either an active functional foot orthosis (FFO A) group or a sham orthosis group. On completion of the feasibility trial, to explore further the mechanism of action, participants in the sham group were given the option to try an alternative off-the-shelf FFO (FFO B) (see Supplementary Fig. S1).

Fig. S1 .

**Fig. 1:**
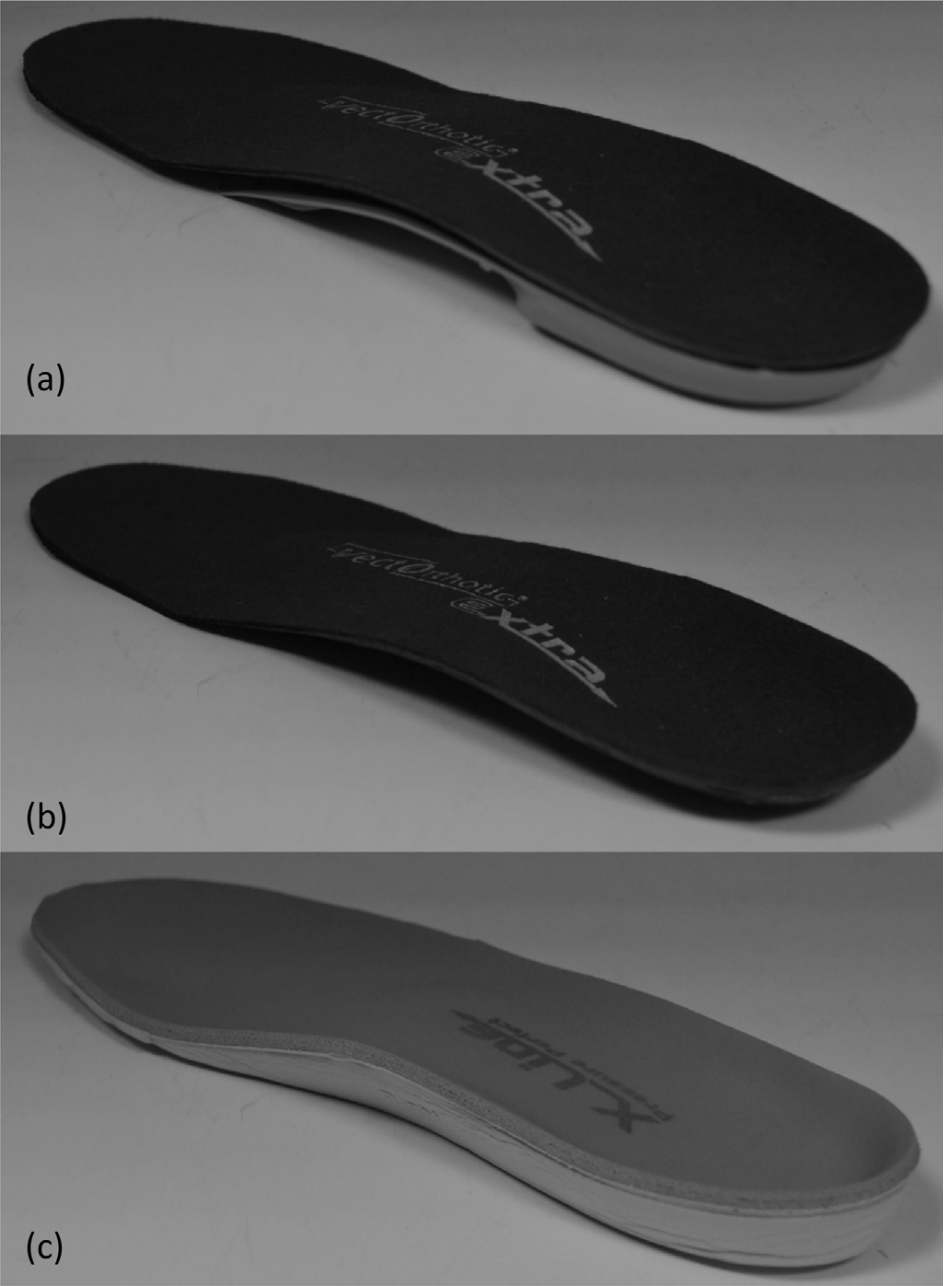
Diagram illustrating the posterior-medial view of the (a) FFO A, (b) Sham orthoses and (c) FFO B.

FFO A is a modifiable off-the-shelf orthotic device (VectOrthotic^®^, Healthy Step [Sensograph] Ltd UK, see Fig. 1a) consisting of a composite polypropylene plastic shell with a contoured arch and heel cup. The shell was modified, where clinically indicated (using hindfoot wedging) to optimise the potential functional effect of the device on the medial midfoot region (for more details on orthoses modification see [22]). FFO A was finished by adding a 4 mm compressed closed cell polyethylene foam cover with a brushed nylon top. The sham orthosis comprised only the top cover of the FFO A, thus similar in appearance to FFO A as possible (see Fig. 1b).

FFO B (Pressure Perfect^®^ Healthy Step [Sensograph] Ltd UK]) consisted of a contoured full length orthosis comprising of a 6 mm closed cell EVA foam base (see Fig. 1c) with a heel cup, an arch support and a metatarsal dome contoured into the base structure with a 3.2 mm polyurethane top cover. To minimise the confounding effect of different shoe types, a standardised shoe was worn by each participant during the data acquisition as described previously [22].

### 2.2 Procedures

At the 12 week follow-up appointment, plantar pressure measurements were captured using the Pedar^®^ in-shoe measurement system acquiring at 50 Hz (Pedar, Novel Gmbh, Munich, Germany). Participants walked under two experimental conditions; 1) shoe only and 2) shoe plus their assigned orthoses, in a randomised order and participants were blind to their allocated intervention. Sham group participants who opted to try FFO B completed a third experimental walk (shoe plus FFO B) at the end of the testing session. For each experimental condition, the Pedar^®^ insole was placed on top of the assigned orthoses and the combination inserted into the shoe. Each participant completed three laps of a 10 m walkway at a self-selected speed for each experimental condition. For analysis purposes, between 12 and 16 mid-lap steps were obtained per participant per experimental condition and averages were calculated.

### 2.3 Data analysis

**Fig. 2:**
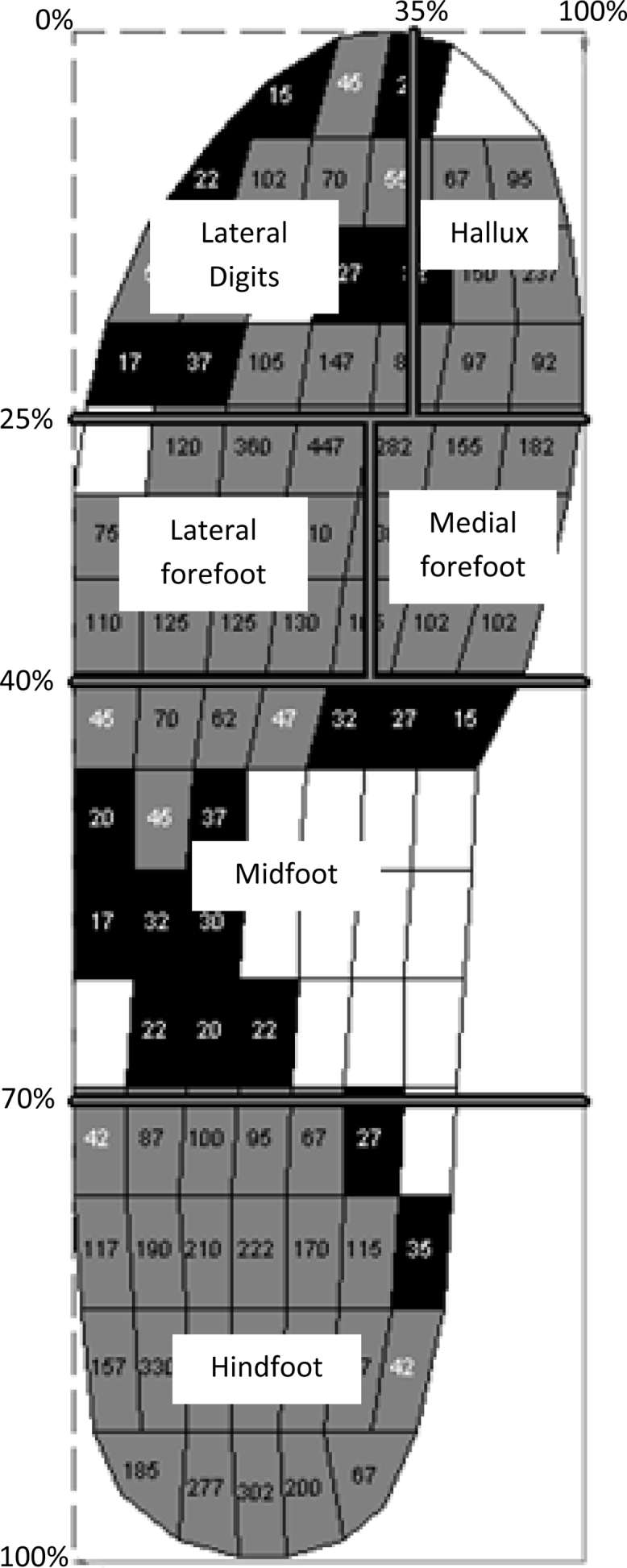
The masked regions of the foot defined by the percentage mask. Note: that the Hallux and lateral digits were defined but not included in the analysis.

Maximum force (% of body weight [BW]), peak pressure (kPa), contact area (cm^2^) and contact time (% roll over process [ROP]) under the different regions on the foot were extracted for analysis. These specific variables were selected *a priori*, based on theories of function and existing literature, to avoid data mining while attempting to explain the mechanism(s) of action of the FFOs and sham compared to the shoe only condition. Plantar pressure data were analysed using Novel-win program (version 0.8 Novel Win GmbH, Munich) and Novel percent mask was applied by dividing the affected foot into six regions (see Fig. 2). Hallux and lesser toes regions were excluded from the analysis, having previously yielded relatively high variability [24] and limited importance for measuring the effects of orthoses on midfoot OA.

This was a secondary exploratory analysis rather than a hypothesis driven study, and so formal inferential analysis is inappropriate. Results are therefore presented descriptively as mean differences and 95% confidence intervals (CI) between orthosis condition minus shoe only condition, performed using IBM SPSS Statistics, version 19.

## 3 Results

**Fig. 3:**
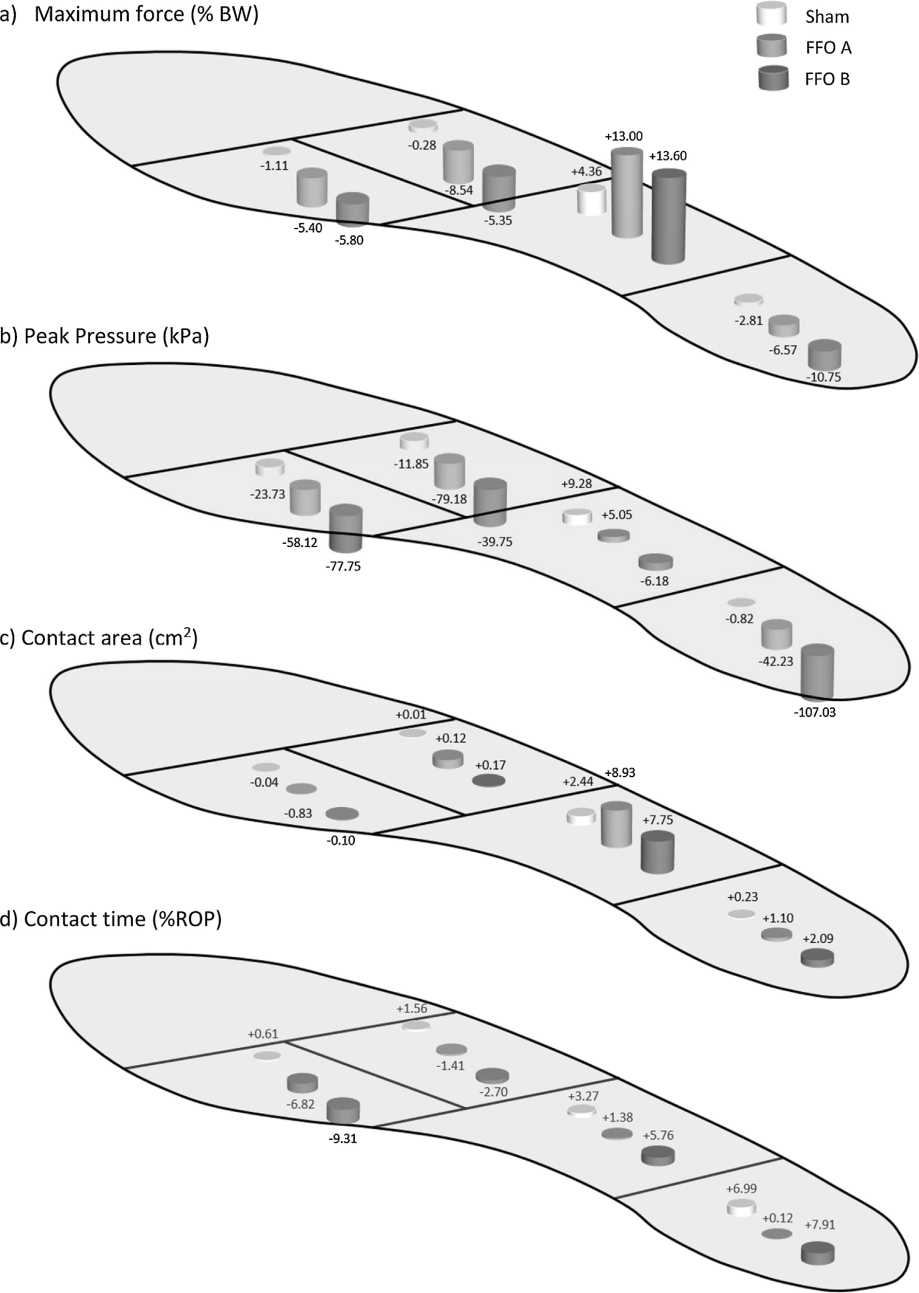
Differences from shoe only condition for a) Maximum force, b) Peak Pressure, c) Contact area and d) Time (as a % of rollover) associated with wearing the sham orthoses (white cylinder), FFO A (light grey cylinder) and FFO B (dark grey cylinder).

Thirty-three participants (23 female), aged 61.0 ± 11.5 (mean ± SD), BMI 29.5 ± 4.2 kg/m^2^, with symptomatic midfoot OA completed the 12 week feasibility trial [22]. Midfoot OA was reported in a median of two joints (range 1–4) at the cuneiform-second metatarsal joint (82%), followed by the naviculo-medial cuneiform joint (70%), the cuneiform-first metatarsal joint (48%) and the talo-navicular joint (30%). Table 1 shows the demographics and clinical characteristics for each group. Eighteen participants were assigned to wear FFO A and 15 assigned to the sham orthoses, of which 14 of the 15 chose to participate in the additional testing of FFO B. Fig. 3a–d illustrates the mean difference from the shoe only condition associated with wearing FFO A, FFO B or sham orthoses for each chosen variable. Results from the full analysis are presented in Supplementary Table S1, Table S2, Table S3, Table S4 for each masked region.

Table S1 .

Table S2 .

Table S3 .

Table S4 .

### 3.1 Hindfoot

Both FFOs increased contact area (FFO A = 1.10 cm^2^, 95% CI 0.55 to 1.64; FFO B = 2.09 cm^2^, 95% CI 0.86 to 3.33) under the hindfoot whilst decreasing maximum force (FFO A = −6.57% BW, 95% CI −9.77 to −3.75; FFO B = −10.75% BW, 95% CI −14.56 to −6.94) and consequently decreasing peak pressure (FFO A = −42.23 kPa, 95% CI −70.33 to −14.24; FFO B = −107.03 kPa, 95% CI −143.09 to −70.97). FFO A had no effect on contact time compared to the shoe only condition. FFO B increased contact time (7.91% ROP) under the hindfoot, however the effect was variable (CI 0.42 to 15.40).

Compared to the shoe only condition, the sham orthoses increased contact time (6.99% ROP, 95% CI 0.41 to 13.56). There was a small decrease in maximum force (2.81% BW, 95% CI −6.31 to 0.68) but wide CI suggests little systematic difference between conditions. The sham orthoses had a minimal effect on contact area or peak pressure under the hindfoot compared to the shoe only condition (see Supplementary Table S1).

### 3.2 Midfoot

Compared to the shoe only condition, both FFOs increased maximum force (FFO A = 13.00% BW, 95% CI 10.37 to 15.63; FFO B = 13.60% BW, 95% CI 9.84 to 17.36) and contact area (FFO A = 8.93 cm^2^, 95% CI 7.03 to 10.82; FFO B = 7.75 cm^2^, 95% CI 4.53 to 10.98) under the midfoot. Overall, midfoot contact time increased with the addition of FFO A and B although the effect was variable (see Supplementary Table S2) suggesting no evidence of a systematic effect. FFO A caused a small increase whereas FFO B decreased peak pressure compared to the shoe only condition. However, for both FFOs, the CI were wide and crossed zero (see Supplementary Table S2).

The sham orthoses increased contact area (2.44 cm^2^, 95% CI 0.27 to 4.61) and maximum force (4.36%, 95% CI 1.67 to 7.05) under the midfoot compared to shoe only. The sham orthoses increased peak pressure and contact time under the midfoot; however CI for both measures crossed zero suggesting a similar effect to the shoe only condition (see Supplementary Table S2).

### 3.3 Medial forefoot

Both FFOs reduced maximum force, peak pressure and contact time (see Supplementary Table S3). FFO A caused a small decrease in contact area of the medial forefoot compared to the shoe only condition whereas the effect of FFO B was minimal. For all plantar pressure measures, there were small differences between the sham orthoses and shoe only conditions with CIs crossing zero.

### 3.4 Lateral forefoot

Both FFOs caused reductions in maximum force and peak pressure. Contact area and contact time were similar under the lateral forefoot when wearing either FFO compared to the shoe only condition, with CI crossing zero (see Supplementary Table S4). The sham orthoses produced similar plantar pressure outcomes to the shoe only condition, with wide CIs for all measures.

## 4 Discussion

This exploratory study aimed to compare the mechanical effects of two off-the-shelf FFOs and a sham orthosis, relative to a baseline shoe-only condition, in order to determine the plausibility of utilising either FFO as a potential treatment for midfoot OA. Findings demonstrate that both FFOs produce similar mechanical effects across the entire foot-orthosis interface, whilst the sham orthoses compared favourably to the shoe only condition.

Both FFOs had similar mechanical effects in most regions of the foot. Under the hindfoot, both FFOs increased contact area whilst decreasing maximum force and peak pressure, with FFO B exhibiting these findings to a greater extent compared to FFO A. Although FFO B caused greater contact time and area under the hindfoot, this trend was more variable, with wider CI for both measures, suggesting differences between the two FFOs is relatively unimportant. The observed effects may reflect the greater thickness and cushioning material used in the construction of FFO B compared to FFO A.

Compared to the shoe only conditions, both FFOs resulted in maximum force being redistributed systematically from the hindfoot and forefoot regions towards the midfoot. This increase was combined with an increase in midfoot contact area and therefore had minimal effect on midfoot peak pressure, suggesting the effect is due to the closely fitted contoured arch support of both FFOs which is consistent with past research [24], [25], further supporting the notion that contoured orthoses act as a fulcrum at the midfoot, thus prolonging loading in this region of the foot [26]. This is pertinent because the 2nd cuneometatarsal joint has been shown previously to be the most commonly affected joint in midfoot OA [10], [14], [15]. People with midfoot OA have also been shown to have anatomical differences at the midfoot and have greater loads acting on the midfoot compared to those without midfoot OA [15], [16]. Halstead et al. [22] demonstrated that wearing FFO A for 12 weeks, significantly improved patient-reported pain and function compared to a sham orthosis and the present results suggest a potential mechanism of action for its clinical effect. These new findings suggest that FFO A is a plausible treatment for symptomatic midfoot OA, reducing pain via a demonstrable mechanical effect. Whether these orthoses result in long-term improvements in midfoot pain or loading requires further investigation.

The active FFO interventions caused similar mechanical effects under the medial and lateral forefoot. Both FFOs caused a systematic decrease in maximum force, peak pressure and contact time. These findings are consistent with past research [24], [25] and support the notion that there are generic properties within the family of contoured off-the-shelf foot orthoses that may provide mechanical benefits for those with forefoot complaints. Future research is required to test this hypothesis. The precise mechanism of action of the FFOs remains more complex than that demonstrated simply at the orthoses-foot interface, but our current findings suggest the mechanical effect of either FFO, and their influence in reducing the pain of midfoot OA may arise though a combination of direct splinting of the region through the close fitting orthotic shell, together with a reduction in loads applied to either end of the complex jointed anatomy, with the corresponding reduction in bending moments across affected joints.

### 4.1 The mechanical effect of the sham device compared to a standard shoe

Recently there has been increasing interest around identifying an appropriate control or sham condition for studies examining FFO on lower limb OA [23], [27], [28], [29]. McCormick et al. [29] and Felson et al. [23] suggested studies that incorporate a sham device should explicitly quantify the mechanical effect of the orthoses. In the present study, the sham device comprised the top cover portion of the FFO A and therefore very similar in appearance to the active intervention, while testing the hypothesis that the sham would have minimal mechanical effects. The study confirmed the sham orthoses had a minimal effect on the hindfoot and both forefoot regions compared to the shoe only condition therefore can be considered an appropriate sham. At the midfoot, the sham orthoses increased contact area (2.44 cm^2^) and maximum force (4.36% BW) compared to the shoe only condition. Although this was less than one-third of the effect seen with the active FFOs, it indicates some mechanical effect is associated with this specific top cover when used as a sham. The effect of increasing midfoot maximum force and contact area may result from the 4 mm thickness of the top cover material. The small increase in maximum force in the sham condition in the present study is consistent with previous findings that demonstrated that wearing a similar sham device for 4 weeks (contoured 1 mm polyethylene foam insole) increased maximum force under the medial and lateral midfoot by approximately 10% BW, compared to a shoe-only baseline [29]. Based on the present findings, it is clear that there is a small effect of the sham insole on midfoot foot-orthoses interface, which could be argued to have minimal clinical significance. However, this would depend on foot type and whether there was contact between the medial arch and sham insole. When designing future trials examining FFOs, it is important to evaluate the significance of a sham device over a control shoe condition [27], [28]. For future midfoot OA trials, further work is required to consider minimising the mechanical effect at the midfoot whilst maintaining the appearance of a FFO.

There are a number of limitations associated with this study. There are possible measurement errors when using the Pedar insoles on curvilinear insoles as the insoles are calibrated when insoles are placed flat on a hard surface [29]. Pedar insoles are widely used for collecting in-shoe plantar pressures, however there are no commercial systems currently capable of collecting in-shoe pressure and force data that account for curved surfaces. Another limitation of the study is that the wearing in protocol for the FFO B group was different to that for the FFO A and sham groups. Although it has been shown previously there are minimal differences in plantar pressure measures across the foot-orthoses interface when tested at baseline and 4 weeks later [29], ideally, a three armed, 12-week intervention study would have been more appropriate. We note however that the primary comparison of FFO A versus sham has been further supported by the comparability of the data for FFO B when this group was added.

## 5 Conclusions

Functional foot orthoses have been shown to provide short-term (12 weeks) clinical benefit in patients with midfoot OA. To our knowledge, this is the first study to compare against sham, the mechanical properties of two differently designed off-the-shelf FFOs for the treatment of midfoot OA. Both FFOs yielded similar mechanical effects across the entire foot-orthoses interface and suggest that both FFOs are plausible conservative treatments for midfoot OA patients. The sham insole exhibited minimal influence on the forefoot and hindfoot but did increase loading in the midfoot region, albeit much less than the active devices. Further work is required to understand the trade-off between convincing appearance and mechanical effects of sham FFOs. In the interim the current study quantifies the extent to which a sham such as used here might influence midfoot mechanical function and allows this to be factored into the planning of future intervention studies.

## Conflict of interests

None

## Role of the funding source

No funding source played any role in the design or conduct of this study or in the writing on the manuscript.

## Authors contributions

ACR and JH conceived of the study idea and designed the study. JH collected and processed the data. GJC, JH and ACR analysed and interpreted the data. GJC drafted the manuscript. All authors revised the manuscript for intellectual content and approved of the final article prior to submission.

## Figures and Tables

**Table 1:** Demographics and clinical characteristics of participants.

	Functional foot orthoses A (n = 18)	Functional foot orthoses B (n = 14)	Sham intervention (n = 15)
Age (years)	61.7 (9.1)	59.8 (14.6)	60.3 (14.2)
Gender (Female)	14 (77.8%)	8 (57.1%)	9 (60.0%)
Body Mass Index (kg/m^2^)	30.8 (4.3)	28.2 (3.9)	28.0 (3.9)
Right foot affected (proportion)	10 (55.6%)	6 (42.9%)	7 (46.7%)

Values are reported as mean (SD) unless otherwise stated.
