# Supplementary material for: Comparability of off the shelf foot orthoses in the redistribution of forces in midfoot osteoarthritis patients
Source: Gait Posture. 2016 Sep;49:235–40. doi: 10.1016/j.gaitpost.2016.07.012 (PMC5038933; doi:10.1016/j.gaitpost.2016.07.012)
Supplement: Fig. S1 — . [file mmc5.docx]

**Supplementary Table 4**. Means (SD) between orthoses condition and mean change shoe only - orthoses condition (95% confidence intervals) for the lateral forefoot

| **Lateral forefoot** | | | | | | | | | | | | | | | | |
| --- | --- | --- | --- | --- | --- | --- | --- | --- | --- | --- | --- | --- | --- | --- | --- | --- |
|  | | **Mean (SD)** | | | **Mean difference**  **(95% CI)** | | **Mean (SD)** | | | **Mean difference**  **(95% CI)** | | **Mean (SD)** | | | **Mean difference**  **(95% CI)** | |
|  | **Shoe only**  **(n=15)** | | **Sham**  **(n=15)** | **Sham Orthosis – shoe only** | | **Shoe only**  **(n=18)** | | **FFO A (n=18)** | **FFO A – shoe only** | | **Shoe only**  **(n=14)** | | **FFO B (n=14)** | **FFO B – shoe only** | |  |
| Maximum force (%BW) | 31.32 (12.48) | | 31.03 (12.49) | -0.28  (-2.82 to 2.25) | | 36.85 (10.86) | | 28.31 (10.19) | -8.54  (-12.75 to -4.32) | | 31.44 (12.94) | | 26.08 (10.49) | -5.35  (-8.92 to -1.78) | |  |
| Peak Pressure (kPa) | 246.68 (89.10) | | 234.83 (82.43) | -11.85  (-36.72 to 13.02) | | 298.66 (116.42) | | 219.48 (84.02) | -79.18  (-119.64 to -38.71) | | 248.90 (92.04) | | 209.15 (68.72) | -39.74  (-65.03 to -14.46) | |  |
| Contact area (cm^2^) | 19.73 (2.91) | | 19.72 (2.40) | 0.01  (-0.49 to 0.52) | | 20.06 (2.33) | | 20.18 (2.30) | 0.12  (-0.01 to 0.24) | | 19.70 (3.01) | | 19.86 (2.90) | 0.17  (-0.64 to 0.97) | |  |
| Contact time (%ROP) | 94.48 (6.32) | | 96.03 (3.93) | 1.56  (-1.36 to 4.47) | | 95.56 (2.77) | | 94.15 (2.64) | -1.41  (-2.83 to 0.01) | | 94.09 (6.37) | | 91.39 (4.45) | -2.70  (-6.27 to 0.88) | |  |
