# Supplementary material for: Comparability of off the shelf foot orthoses in the redistribution of forces in midfoot osteoarthritis patients
Source: Gait Posture. 2016 Sep;49:235–40. doi: 10.1016/j.gaitpost.2016.07.012 (PMC5038933; doi:10.1016/j.gaitpost.2016.07.012)
Supplement: Table S1 — . [file mmc1.docx]

**Supplementary Figure 1**. Consort diagram of the feasibility trial (shaded area as reported by Halstead [22]) with the additional analysis of the present, mechanism of action study (below dashed line).

Assessed for eligibility (n= 119)

Excluded (n= 81)

♦ Not meeting inclusion criteria (n=7)

♦ Declined to participate (n= 8)

♦ Lost to follow-up (n= 1)

Randomised (n=37)

## Analysis

♦ Analysis of sham (n=15)

None excluded from analysis

♦ Analysis of FFO A (n= 18)

None excluded from analysis

## Enrolment

## Follow-Up

♦ Allocated to sham intervention (n=18)

♦ Received allocated intervention (n=18)

♦ Lost to follow-up (n=0)

♦ Discontinued intervention (n=1)

Reason: Pain related to foot orthoses

♦ Allocated to FFO A intervention (n=19)

♦ Received allocated intervention (n=19)

♦ Lost to follow-up (n=1)

♦ Discontinued intervention (n= 2)

Reason: Pain unrelated to foot orthoses

## Allocation

♦ Analysis of FFO B (n=14)

♦ Declined to participate in additional testing (n=1)

[22] Halstead J, Chapman GJ, Gray JC, Grainger AJ, Brown S, Wilkins RA, et al. Foot orthoses in the treatment of symptomatic midfoot osteoarthritis using clinical and biomechanical outcomes: a randomised feasibility study. Clinical rheumatology. 2016;35:987-96.
