# Supplementary material for: Comparability of off the shelf foot orthoses in the redistribution of forces in midfoot osteoarthritis patients
Source: Gait Posture. 2016 Sep;49:235–40. doi: 10.1016/j.gaitpost.2016.07.012 (PMC5038933; doi:10.1016/j.gaitpost.2016.07.012)
Supplement: Table S2 — . [file mmc2.docx]

**Supplementary Table 1**. Means (SD) between orthoses condition and mean change shoe only - orthoses condition (95% confidence intervals) for the hindfoot

| **Hindfoot** | | | | | | | | | | | | | | | | |
| --- | --- | --- | --- | --- | --- | --- | --- | --- | --- | --- | --- | --- | --- | --- | --- | --- |
|  | | **Mean (SD)** | | | **Mean difference**  **(95% CI)** | | **Mean (SD)** | | | **Mean difference**  **(95% CI)** | | **Mean (SD)** | | | **Mean difference**  **(95% CI)** | |
|  | **Shoe only**  **(n=15)** | | **Sham**  **(n=15)** | **Sham – shoe only** | | **Shoe only**  **(n=18)** | | **FFO A (n=18)** | **FFO A – shoe only** | | **Shoe only**  **(n=14)** | | **FFO B (n=14)** | **FFO B – shoe only** | |  |
| Maximum force (%BW) | 73.13 (11.26) | | 70.13 (12.03) | -2.81  (-6.31 to 0.68) | | 68.86 (13.65) | | 62.29 (10.77) | -6.57  (-9.77 to -3.75) | | 73.03 (11.68) | | 62.27 (13.13) | -10.75  (-14.56 to -6.94) | |  |
| Peak Pressure (kPa) | 304.77 (89.96) | | 303.94 (97.13) | -0.82  (-16.58 to 14.93) | | 264.69 (51.25 ) | | 222.46 (47.62) | -42.23  (-70.33 to -14.24) | | 310.44 (90.42) | | 203.41 (48.63) | -107.03  (-143.09 to -70.97) | |  |
| Contact area (cm^2^) | 38.56 (5.22) | | 38.80 (5.66) | 0.23  (-0.84 to 1.29) | | 38.09 (4.44) | | 39.19 (4.60) | 1.10  (0.55 to 1.64) | | 38.60 (5.41) | | 40.69 (5.91) | 2.09  (0.86 to 3.33) | |  |
| Contact time (%ROP) | 91.26 (12.68) | | 98.25 (2.30) | 6.99  (0.41 to 13.56) | | 97.69 (2.70) | | 97.81 (4.45) | 0.12  (-1.84 to 2.08) | | 90.64 (12.92) | | 98.55 (3.24) | 7.91  (0.42 to 15.40) | |  |
