# Supplementary material for: Comparability of off the shelf foot orthoses in the redistribution of forces in midfoot osteoarthritis patients
Source: Gait Posture. 2016 Sep;49:235–40. doi: 10.1016/j.gaitpost.2016.07.012 (PMC5038933; doi:10.1016/j.gaitpost.2016.07.012)
Supplement: Table S3 — . [file mmc3.docx]

**Supplementary Table 2**. Means (SD) between orthoses condition and mean change shoe only - orthoses condition (95% confidence intervals) for the midfoot

| **Midfoot** | | | | | | | | | | | | | | | | |
| --- | --- | --- | --- | --- | --- | --- | --- | --- | --- | --- | --- | --- | --- | --- | --- | --- |
|  | | **Mean (SD)** | | | **Mean difference**  **(95% CI)** | | **Mean (SD)** | | | **Mean difference**  **(95% CI)** | | **Mean (SD)** | | | **Mean difference**  **(95% CI)** | |
|  | **Shoe only**  **(n=15)** | | **Sham**  **(n=15)** | **Sham Orthosis – shoe only** | | **Shoe only**  **(n=18)** | | **FFO A (n=18)** | **FFO A – shoe only** | | **Shoe only**  **(n=14)** | | **FFO B (n=14)** | **FFO B – shoe only** | |  |
| Maximum force (%BW) | 22.21 (3.48) | | 26.57 (11.63) | 4.36  (1.67 to 7.05) | | 20.55 (6.98) | | 33.55 (10.21) | 13.00  (10.37 to 15.63) | | 22.09 (9.83) | | 35.70 (10.46) | 13.60  (9.84 to 17.36) | |  |
| Peak Pressure (kPa) | 150.30 (63.24) | | 159.58 (59.88) | 9.28  (-5.02 to 23.57) | | 155.75 (35.95) | | 160.80 (35.27) | 5.05  (-11.71 to 21.80) | | 153.42 (64.42) | | 147.24 (44.40) | -6.18  (-29.00 to 16.64) | |  |
| Contact area (cm^2^) | 32.41 (6.63) | | 34.85 (6.18) | 2.44  (0.27 to 4.61) | | 30.80 (8.23) | | 39.73 (7.34) | 8.93  (7.03 to 10.82) | | 32.72 (6.77) | | 40.47 (6.57) | 7.75  (4.53 to 10.98) | |  |
| Contact time (%ROP) | 91.90 (9.71) | | 95.17 (5.44) | 3.27  (-1.50 to 8.05) | | 96.13 (4.07) | | 97.50 (3.74) | 1.38  (-1.07 to 3.83) | | 91.34 (9.82) | | 97.10 (4.16) | 5.76  (0.23 to 11.29) | |  |
