# Supplementary material for: Comparability of off the shelf foot orthoses in the redistribution of forces in midfoot osteoarthritis patients
Source: Gait Posture. 2016 Sep;49:235–40. doi: 10.1016/j.gaitpost.2016.07.012 (PMC5038933; doi:10.1016/j.gaitpost.2016.07.012)
Supplement: Table S4 — . [file mmc4.docx]

**Supplementary Table 3**. Means (SD) between orthoses condition and mean change shoe only - orthoses condition (95% confidence intervals) for the medial forefoot

| **Medial forefoot** | | | | | | | | | | | | | | | | |
| --- | --- | --- | --- | --- | --- | --- | --- | --- | --- | --- | --- | --- | --- | --- | --- | --- |
|  | | **Mean (SD)** | | | **Mean difference**  **(95% CI)** | | **Mean (SD)** | | | **Mean difference**  **(95% CI)** | | **Mean (SD)** | | | **Mean difference**  **(95% CI)** | |
|  | **Shoe only**  **(n=15)** | | **Sham**  **(n=15)** | **Sham Orthosis – shoe only** | | **Shoe only**  **(n=18)** | | **FFO A (n=18)** | **FFO A – shoe only** | | **Shoe only**  **(n=14)** | | **FFO B (n=14)** | **FFO B – shoe only** | |  |
| Maximum force (%BW) | 22.28 (7.60) | | 21.17 (8.39) | -1.11  (-3.47 to 1.26) | | 20.15 (4.68) | | 14.76 (6.52) | -5.40  (-7.21 to -3.58) | | 22.11 (7.86) | | 16.24 (6.53) | -5.88  (-8.71 to -3.04) | |  |
| Peak Pressure (kPa) | 283.37 (65.62) | | 259.64 (84.12) | -23.73  (-61.88 to 14.42) | | 280.93 (91.45) | | 222.81 (105.30) | -58.12  (-101.28 to -14.95) | | 279.09 (65.88) | | 201.34 (63.74) | -77.75  (-103.13 to -52.37) | |  |
| Contact area (cm^2^) | 11.88 (2.40) | | 11.84 (2.47) | -0.04  (-0.82 to 0.73) | | 11.78 (1.59) | | 10.95 (2.29) | -0.83  (-1.56 to - 0.92) | | 11.89 (2.49) | | 11.79 (2.20) | -0.10  (-1.00 to -0.81) | |  |
| Contact time (%ROP) | 86.53 (7.32) | | 87.15 (9.84) | 0.61  (-3.15 to 4.38) | | 89.27 (7.64) | | 82.45 (11.20) | -6.82  (-10.59 to -3.04) | | 86.35 (7.56) | | 77.04 (12.78) | -9.31  (-14.77 to -3.84) | |  |
